# A Mediterranean-Style Diet Plan Is Associated with Greater Effectiveness and Sustainability in Weight Loss in Patients with Obesity after Endoscopic Bariatric Therapy

**DOI:** 10.3390/medicina58020168

**Published:** 2022-01-22

**Authors:** Lidia Rueda-Galindo, María Fernanda Zerón-Rugerio, Antonio J. Sánchez Egea, Gil Serrancolí, Maria Izquierdo-Pulido

**Affiliations:** 1 Department of Nutrition, Food Science, and Gastronomy, Food Science Torribera Campus, University of Barcelona, 08921 Barcelona, Spain; lrueda.galindo@gmail.com (L.R.-G.); fernanda.zeron@ub.edu (M.F.Z.-R.); 2 Dorsia Clinics Surgery and Aesthetic Medicine, 08008 Barcelona, Spain; 3 Nutrition and Food Safety Research Institute (INSA-UB), University of Barcelona, 08921 Barcelona, Spain; 4 Department of Mechanical Engineering, EEBE, Universidad Politécnica de Cataluña, 08034 Barcelona, Spain; antonio.egea@upc.edu (A.J.S.E.); gil.serrancoli@upc.edu (G.S.)

**Keywords:** endoscopic bariatric therapy, Mediterranean diet, weight loss, obesity

## Abstract

This study aimed to investigate the impact of a Mediterranean-style diet on weight loss effectiveness and sustainability in patients with obesity who underwent endoscopic bariatric therapies (EBT), relative to a protein diet plan. Thus, 132 patients with obesity (BMI 30–40 kg/m^2^) who underwent EBT, were asked to follow a Mediterranean-style diet plan (*n* = 52) or a protein diet plan (*n* = 26) for six months. General linear models were used to compare outcome variables between dietary intervention groups. Results showed that participants who followed a Mediterranean-style diet plan lost 14.2% more weight (95% CI: 3.0; 25.3), compared with those who followed a protein diet plan. Additionally, following a Mediterranean-style diet plan was associated with the sustainability of weight loss. Note that three months after the end of the dietary intervention, the patients who followed a Mediterranean-style diet plan were still losing weight (−1.2 ± 3.0 kg), while those with a protein diet plan gained, on average, 2.4 ± 3.3 kg (*p* < 0.001). Therefore, we conclude that combining EBT with a Mediterranean-style diet plan could represent an effective dietary intervention to improve the effectiveness and sustainability of weight loss after an EBT.

## 1. Introduction

Lifestyle modification is recommended as a cornerstone of obesity treatment. However, many people do not achieve lasting benefits due to difficulty with adherence and to the body’s physiological and neurohormonal adaptations in response to weight loss [1]. Therefore, several medical therapies, such as pharmacotherapy or bariatric surgery, have been proposed for the treatment of obesity [1,2]. Bariatric surgery yields substantial and sustained weight loss over time, remission of comorbidities (primarily type 2 diabetes) and improvement in the quality of life of the patient [2,3]. Nevertheless, it is associated with short- and long-term nutritional deficiencies and procedural complications [1]. Consequently, minimally invasive endoscopic bariatric therapies (EBT) have been developed [1,4], such as the BioEnterics intragastric balloon (BIB) and primary obesity surgery endoluminal (POSE) [4]. Specifically, BIB consists primarily of a space-occupying gastric therapy, where a balloon filled with saline solution is placed and removed endoscopically [4]. Meanwhile, POSE is an endoscopic procedure that involves the plication of the fundus and the distal body of the stomach [4].

Although these EBT are considered effective weight loss therapies [4,5,6], dietary interventions are a cornerstone of obesity treatment. In fact, emerging evidence has shown that adherence to healthy dietary patterns is an essential factor in the success of weight loss over time [2]. The Mediterranean diet is a healthy dietary pattern which has been associated with weight loss in the general population [7,8,9]. However, it is unknown whether combining EBT with a Mediterranean-style diet plan can be more effective in weight loss than classical approaches. Therefore, our objective was to investigate the impact of a 6-month dietary intervention with a Mediterranean-style diet plan on the effectiveness of weight loss in patients with obesity who underwent EBT, compared with a protein diet plan. Furthermore, we analyzed which diet plan (Mediterranean-style or protein) was associated with the sustainability of weight loss three months after the dietary intervention.

## 2. Materials and Methods

The participants were recruited for a six-month study at Dorsia Clinics (Barcelona, Spain) between 2015 and 2018. Recruitment consisted of explaining to the patients the details of the research and inviting them to participate in the study. Eligibility criteria included: age ≥18 years, BMI 30–40 kg/m^2^, no previous gastric intervention, no hiatus hernias, not being pregnant or breastfeeding, having no cardiac problems, regular values in coagulation and blood count, and positive psychological evaluation. Based on these criteria, a total of 132 patients were eligible and provided written informed consent. A total of 54 participants were excluded due to adverse effects of the surgery or because they refused to complete the treatment, resulting in a final analytical sample of 78 patients (88.5% females), see Figure S1.

### 2.1. Ethical Statement

The study was performed in accordance with the ethical guidelines of the Declaration of Human Studies of Helsinki and approved by the Ethics Committee of the University of Barcelona (IRB00003099). Additionally, written informed consent was obtained from all study participants and patient data were coded to maintain anonymity.

### 2.2. Study Protocol

During the first week after the EBT (BIB or POSE), participants followed a liquid diet plan [10]. The patients were then randomly assigned to one of the dietary intervention groups: a Mediterranean-style or a protein diet plan (which is the diet plan that is usually recommended for these patients) that they followed for six months. Energy intake was calculated according to the Spanish Consensus on Bariatric Endoscopy [10], and the percentage of macronutrients (expressed as % of the total energy intake) was as follows. Mediterranean-style diet plan: 22% proteins, 53% carbohydrates, and 25% fats; the protein diet plan: 40% proteins, 29% carbohydrates, and 31% fats.

### 2.3. Outcome Variables

#### 2.3.1. Anthropometric Parameters

All participants were weighed pre-EBT and on each study visit (1, 2, 3, 4, 5 and 6 months after the intervention), wearing light clothes to the nearest 0.1 kg, using a body composition analyzer (TANITA C-240MA, Tokyo, Japan). Height was determined during the first study visit using a fixed wall stadiometer (SECA 217, Seca, Hamburg, Germany) to the nearest 0.1 cm, in a standing position. Body mass index (BMI) was calculated as weight (kg) divided by height squared (m^2^).

#### 2.3.2. Weight Loss Effectiveness

Weight loss effectiveness was evaluated as a function of postoperative weight loss as follows [11]:

i. Total weight loss (%TWL) = (Weight loss/Initial weight) ∗ 100;
ii. Excess weight loss (%EWL) = ((Initial weight − Postoperative weight)/(Initial weight − Ideal weight)) ∗ 100.

Note that excess body weight was calculated based on a reference body weight (also known as “ideal body weight”) of BMI 25 kg/m^2^.

#### 2.3.3. Sustainability of Weight Loss

To assess the sustainability of weight loss, the patients were weighed three months after finishing the dietary intervention. We then calculated weight relapse as a marker of the sustainability of weight loss as follows:

i. Weight relapse (kg) = Weight (kg) 3 months after the intervention − Weight (kg) after 6 months of EBT.

### 2.4. Statistical Analyses

Normality of the data was confirmed in all variables using histograms and Q-Q plots. Variables are described as mean and standard deviation (unless stated otherwise). General linear models (GLMs) were used to compare differences in age, initial BMI, and %TWL between dietary intervention groups (Mediterranean-style or protein diet plan). Additionally, chi-square tests were used to test differences in gender and type of EBT between groups. We the used GLMs to compare %EWL and weight relapse between dietary intervention groups. All analyses were performed using SPSS Statistics v25 and adjusted for age, gender, and type of EBT.

## 3. Results

Briefly, a total of 78 patients (88.5% women) with obesity who underwent EBT completed a 6-month dietary intervention with either a Mediterranean-style diet plan (*n* = 52) or a protein diet plan (*n* = 26) (Figure S1). Regarding the general characteristics, we observed that age, gender, and type of EBT were similarly distributed between the dietary intervention groups (Table 1). Likewise, initial BMI was similar between patients who followed a Mediterranean-style or a protein diet plan (*p* = 0.543). Despite these similarities, our data showed that patients who followed a Mediterranean-style diet plan lost more weight (expressed as %TWL) six months after EBT, compared with those who followed the protein diet plan (18.7% ± 4.5 vs. 13.5% ± 5.0; *p* < 0.001).

**Figure 1:**
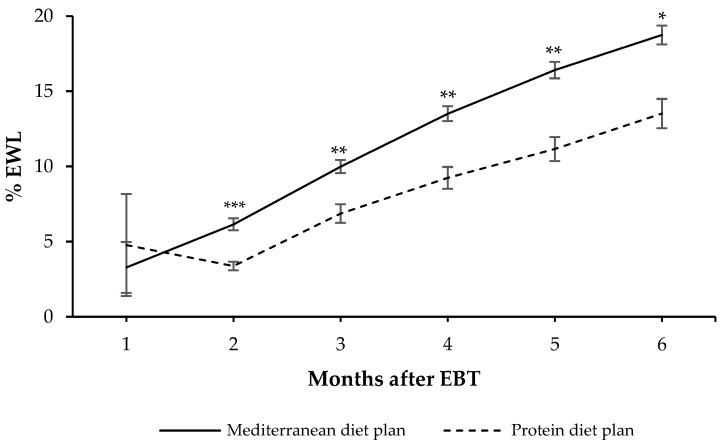
Effectiveness of weight loss expressed as percentage of excess weight loss (%EWL) six months after the endoscopic bariatric therapy (EBT) according to the diet plan followed. Values are expressed as mean and standard error measure. General linear models adjusted for age, gender and type of EBT were used to compare differences in weight loss between groups. * *p* < 0.05, ** *p* < 0.01, and *** *p* < 0.01.

In addition, we observed significant differences in weight-loss effectiveness (expressed as %EWL) between patients who followed a Mediterranean diet plan versus those who followed a protein diet plan (Figure 1). Specifically, our data revealed that from the second to the sixth month after the EBT, %EWL was significantly higher among patients following a Mediterranean-style diet plan, compared with those following a protein diet plan (Figure 1). Note that after six months of treatment with EBT, patients under a Mediterranean-style diet plan lost 14.2% more EWL [95% CI: 3.0; 25.3] than those who followed the protein diet plan.

**Figure 2:**
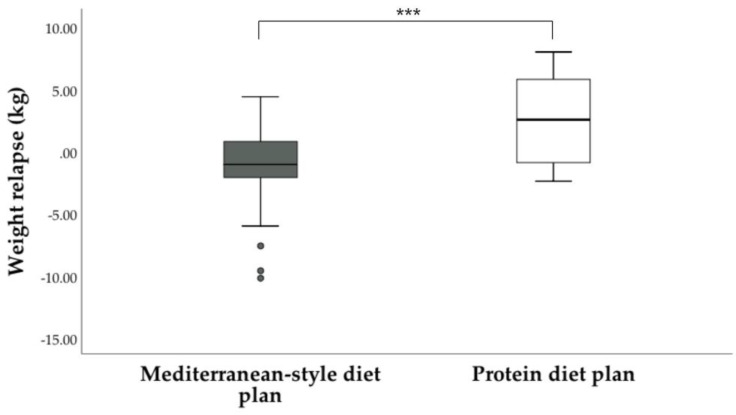
Weight relapse 3 months after completing the dietary intervention. General linear models adjusted for age, gender and type of EBT were used to compare weight relapse between groups. *** *p* < 0.001.

Regarding the sustainability of weight loss (Figure 2), we observed that the participants who followed a Mediterranean-style diet plan continued to lose weight three months after the end of the intervention (−1.2 ± 3.0 kg), while those who followed a protein diet plan gained, on average, 2.4 ± 3.3 kg (*p* < 0.001). Additionally, we noted a greater dispersion of weight relapse was observed in patients who followed a protein diet plan compared with those who followed a Mediterranean-style diet plan.

## 4. Discussion

To the authors’ knowledge, this is the first research study to address the impact of a Mediterranean-style diet plan in the effectiveness and sustainability of weight loss in a sample of patients with obesity who underwent EBT. Note that, six months after the intervention, patients who followed the Mediterranean-style diet plan lost 14.2% more EWL compared to those who followed a protein diet plan. In addition, we showed that three months after completing the dietary intervention, patients who followed a Mediterranean-style diet plan continued to lose weight (−1.2 kg), while those who followed the protein diet plan actually gained weight (~2.4 kg).

The findings of this study are based on the fact that the Mediterranean diet is attractive and realistic enough for the patients to adhere to [12]. Importantly, this dietary pattern is characterized by an abundance of plant-based foods, a moderate intake of fish and dairy, a low intake of red meat, and the use of extra virgin olive oil as the main source of dietary fat [8,13,14]. It has been demonstrated that, despite its relatively high fat content, the high content of antioxidants and anti-inflammatory components in the Mediterranean diet has been shown to promote weight loss [8,14]. Subsequently, patients who underwent EBT and followed a Mediterranean-style diet plan lost more weight six months after the intervention, and also had less weight relapse.

Along these lines, our results showed that the Mediterranean-style diet plan was associated with the sustainability of weight loss. It should be noticed that three months after completing the treatment, patients following the Mediterranean diet continued to lose weight (−1.2 kg). Meanwhile, greater weight regain was observed among participants following a protein diet plan (~2.4 kg). The latter could be attributed to several reasons, including the introduction of new types of food after completing a restrictive diet plan, and anxiety experienced when introducing new foods into their diet [7,9]. Therefore, the type of dietary intervention is important to help patients with obesity achieve their goals and also to encourage them to continue losing weight [7,9], even after the nutritional counseling has ended.

Our study has certain limitations when interpreting our findings. First, we acknowledge that our sample consisted mostly of women, which is not representative of the entire population. Additionally, we acknowledge the small sample size as a limitation of the study. However, this is the first study to investigate the impact of a Mediterranean-style diet plan in the effectiveness and sustainability of weight loss in a sample of patients with obesity who underwent EBT.

## 5. Conclusions

Our findings suggest that a Mediterranean-style diet plan is associated with greater weight loss effectiveness in patients with obesity who underwent EBT. Furthermore, this dietary pattern was related to the sustainability of weight loss, which is a crucial aspect of any weight loss treatment. These results open a new framework in terms of dietary interventions for patients undergoing EBT. However, future studies are needed to support our findings, and to evaluate the impact of a Mediterranean-style diet plan on body composition, as well as other metabolic markers.

## Figures and Tables

**Table 1:** General characteristics of the population studied.

	Protein Diet Plan (*n* = 26)	Mediterranean Diet Plan (*n* = 52)	*p*-Value
Age, years	38.7 (12.2)	43.1 (11.9)	0.132
Gender, % female	22 (84.6)	48 (92.4)	0.095
Type of EBT			
BIB, %	16 (61.9)	37 (72.2)	0.235
POSE, %	10 (38.1)	15 (27.8)	
Initial BMI, kg/m^2^	36.5 (5.0)	37.2 (4.6)	0.543
Total weight loss, %	13.5 (5.0)	18.7 (4.5)	**<0.001**

BMI, body mass index; BIB, BioEnterics intragastric balloon; EBT, endoscopic bariatric treatment; POSE, primary obesity surgery endoluminal. Values are presented as mean (SD) or number (%). Statistical tests: Student’s *t*-test for continuous variables and the chi-squared test for categorical variables. Significant *p*-values are shown in bold.

## Supplementary Materials

The following are available online at https://www.mdpi.com/article/10.3390/medicina58020168/s1, Figure S1: CONSORT flow diagram of participants.
