# Supplementary figures and images for: A Mediterranean-Style Diet Plan Is Associated with Greater Effectiveness and Sustainability in Weight Loss in Patients with Obesity after Endoscopic Bariatric Therapy
Source: Medicina (Kaunas). 2022 Jan 22;58(2):168. doi: 10.3390/medicina58020168 (PMC8875593; doi:10.3390/medicina58020168)

Figure S1. CONSORT flow diagram of participants

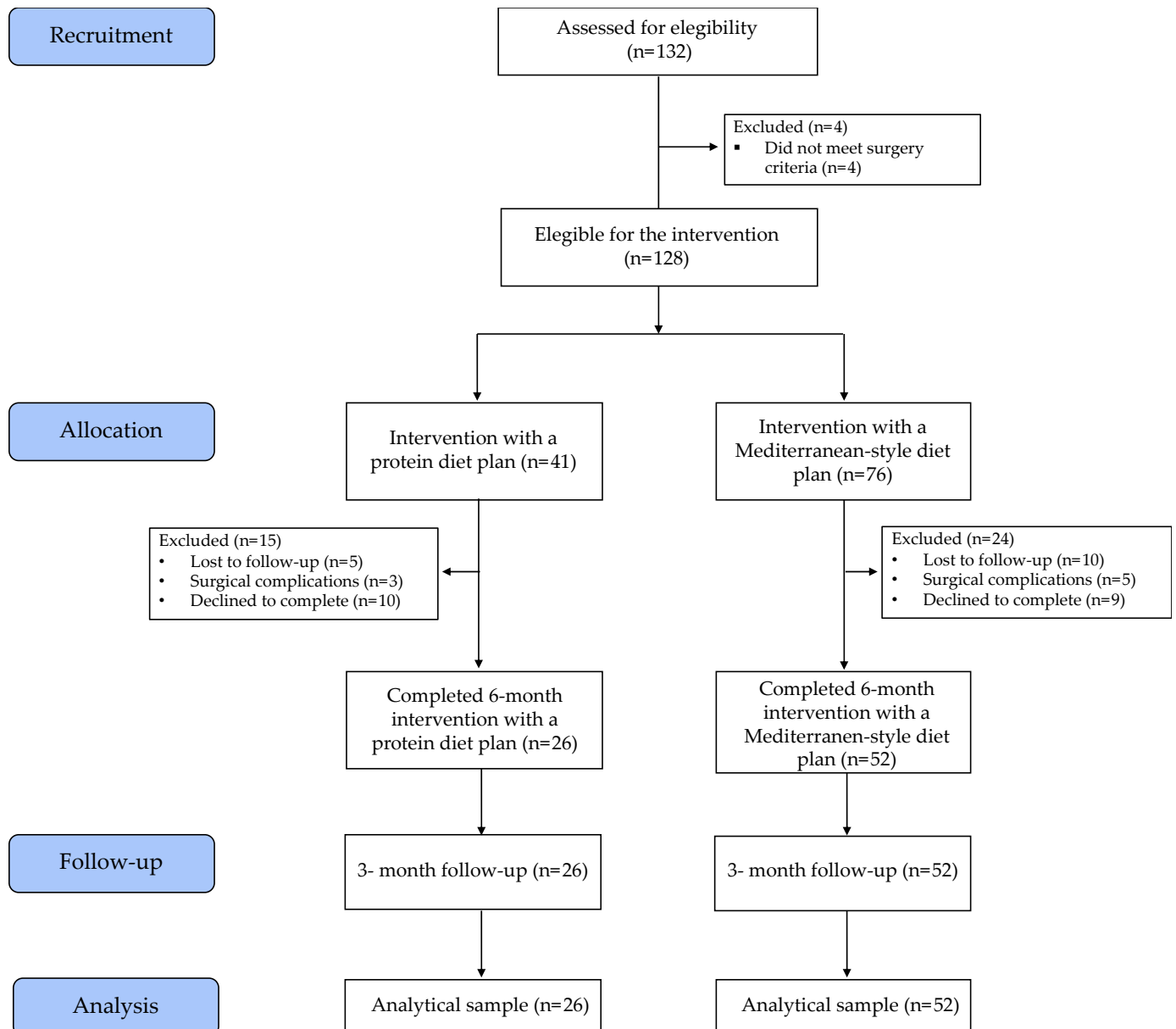

Supplement: Supplementary file 1 [file medicina-58-00168-s001.zip › medicina-1536710-supplementary.pdf]
